# Differences Between Esophageal and Tracheal Intubation Ultrasound View Proficiency: An Educational Study of Novice Prehospital Providers

**DOI:** 10.7759/cureus.8686

**Published:** 2020-06-18

**Authors:** Ann Kaminski, Nkechi O Dike, Kerry Bachista, Michael Boniface, Conrad Dove, Leslie V Simon

**Affiliations:** 1 Emergency Medicine, Mayo Clinic, Jacksonville, USA; 2 Emergency Medicine/Clinical Anatomy, University of Cape Coast, Cape Coast, GHA; 3 Emergency Medicine, Baptist Medical Center, Jacksonville, USA; 4 Emergency Medicine, Mayo Clinc, Jacksonville, USA; 5 Neurosurgery, Mayo Clinic, Rochester, USA; 6 Emergency Medicine/Medical Simulation, Mayo Clinic, Jacksonville, USA

**Keywords:** upper airway ultrasound, intubation, medical education, procedural education, resuscitation, critical care, ultrasound in critical care, emergency medicine physician, esophageal intubation, paramedic emergency medical services (ems) flight paramedicine

## Abstract

Objectives

Airway ultrasound is now possible in the prehospital setting due to advances in ultrasound equipment portability. We questioned how well prehospital providers without prior experience could determine both esophageal and tracheal placement of an endotracheal tube in cadavers after a brief training course in ultrasound.

Methods

This educational prospective study at the Simulation Center in Mayo Clinic Jacksonville Florida enrolled 50 prehospital providers. Demographic and practice background information was obtained through surveys. Each participant performed a baseline ultrasound to determine endotracheal tube placement in a cadaver that was randomly assigned to an esophageal or tracheal intubation. Participants then repeated the randomized testing after a 15-minute tutorial. Before and after overall accuracy as well as proportions of correct identification of esophageal and tracheal intubations were determined and compared using standard binomial proportion and McNemar’s tests.

Results

None of the participants had prior experience of performing airway ultrasound. Baseline group scores were 60% (CI 45%-74%) for overall accuracy (n=50), 55% (CI 32%-76%) for correct identification of an esophageal intubation, and 64% (CI 44%-81%) for correct tracheal detection. Baseline scores were not significantly different from standard binomial distributions. Post-test scores were 82% (CI 69%-91%) for overall accuracy, 96% (CI 80%-100%) for esophageal intubation detection, and 66.7% (CI 45%-84%) for tracheal intubation detection, with corresponding binomial p-values of <0.001, <0.001, and 0.15. P-values for McNemar’s paired test for combined overall accuracy, correct esophageal detection, and correct tracheal detection were 0.04, 0.02, and 0.62, respectively.

Conclusions

Prehospital participants without prior ultrasound experience demonstrated significant gains in airway ultrasound proficiency after a limited introductory course. Post-training score increases were largely due to a notable increase in correct esophageal intubation detection rates. Learners did not make significant progress in correctly identifying a tracheal intubation. Airway ultrasound educational design may benefit from added emphasis on the potentially more difficult to recognize tracheal intubation view.

## Introduction

Ultrasound (US) imaging of the neck by an experienced operator is supported in the 2015 American Heart Association Guidelines for Cardiopulmonary Resuscitation and Emergency Cardiovascular Care Update as a method to assess endotracheal tube (ETT) placement position after endotracheal intubation [1]. Existing evidence suggests that proficiency in this US skill may be achieved rapidly among physicians and may be of value in prehospital settings [2-6]. Endotracheal intubation is a common critical procedure performed by prehospital providers. Few studies to date have examined the ability and performance of prehospital providers to determine ETT placement with US [7,8].

Current methods to determine ETT placement in the prehospital setting include direct visualization of the ETT passing through the vocal cords, use of physical exam findings (e.g. bilateral breath sounds), and digital and color capnometry measurements. However, multiple factors can result in indeterminate findings, including prolonged cardiac arrest, morbid obesity, facial trauma, and profuse bleeding or vomiting [9]. It is possible that prehospital US for identification of ETT placement may be of value in these situations. Prehospital providers are part of a sample of a broader population of non-physician health care providers responsible for airway management. In resource-limited settings, worldwide portable and standard US equipment may be more accessible than standard confirmatory tools, including disposable or digital capnometry. Providers in these settings often lack the necessary training to implement many US techniques [9,10]. Airway US education for US novices is becoming increasingly more applicable to broader groups of providers practicing critical care.

Our study objectives were to determine if prehospital providers with little to no US experience could achieve notable gains in overall intubation placement accuracy after a brief training course, and if there was a difference in educational gains between the tracheal and esophageal views.

## Materials and methods

Participants and study design

This prospective educational study was deemed exempt by the Mayo Clinic Institutional Review Board. Eligible participants included practicing emergency medical technicians and paramedics as well as trainees about to enter either field. Two local county fire and rescue units as well as local private transport companies were invited to participate. Study staff recruited potential participants through announcements at local emergency medical service (EMS) meetings. The study enrollment period took place from November 2018 to April 2019 at the procedural skills lab in the Simulation Center at Mayo Clinic Jacksonville, Florida. Four formalin-fixed cadavers were selected for use after screening by study staff for obvious abnormalities including tumor or prior procedural intervention that would alter normal sonographic appearance. 

Each participant reported demographic information, including their clinical background, number of years of practice, as well as intubation and US experience. They also rated how useful they perceived US could be in their practice on a Likert scale of 1 to 10, with 10 being extremely useful. Participants were then asked to use US to identify an esophageal versus tracheal intubation in a cadaver that was intubated prior to testing. Intubation was performed by an emergency physician using a glidescope for visualization. Tube placement was randomly generated to achieve an evenly proportioned group of tracheal and esophageal intubations. All sonographic examinations were performed on Sonosite M-Turbo machines (Fujifilm, Bothell, WA) using a HFL38 transducer. Participants were allowed to move the external end of the tube slightly to visualize ETT motion, as dynamic motion has been suggested to improve US visualization of the ETT [8]. Participants were observed during the assessment to ensure that motion did not dislodge the tube from its predetermined location. Participants were not given feedback after the initial assessment. Each participant then received 15 minutes of small group instruction from an emergency medicine physician on the use of linear US to detect ETT placement. The course included a slide show introduction on the use of linear US to identify airway anatomy, followed by identification of the single air-mucosa interface for tracheal tube confirmation, and the “double tract” sign suggestive of esophageal intubation. The cadavers were again intubated using random assignment, and each learner attempted to determine the location of the ETT with US after training.

The participants were asked to identify the ETT placement as either esophageal or tracheal during their assessments. Their results were scored as correct or incorrect. All participants received feedback on their scores at the study conclusion. 

Data collection and analysis

Overall accuracy as well as correct esophageal and tracheal intubation detection accuracies were determined with CIs. Before and after scores from each group were compared using a standard binomial distribution test, and McNemar’s test was performed on all randomly paired data. The Wilcoxon rank-sum test was used to compare before and after perceived usefulness Likert scores. A p-value <0.05 indicated statistical significance in all tests. All data were prepared and analyzed using Microsoft Excel (Redmond, WA) and R software (R-Foundation, open source).

## Results

A total of 50 participants completed the study, including 46 prehospital providers and four prehospital trainees (Table 1). Three participants were women and 47 were men. None of the participants had airway US experience, and none were currently using US in their practice. Five had received prior brief US education in peripheral intravenous line placement, and two had received introductory critical care US instruction. The number of years of prehospital experience was 1-41 years, with a median of 5.5 years. The number of intubations in the past year varied from 0 to 20 with a median of 2. Total career intubations ranged from 0 to 100 with a median of 15.

**Table 1:** Baseline characteristics of participants

Learner type	n (%)
Emergency medical technician	8 (16)
Paramedic	38 (76)
Prehospital trainee	4 (8)
Clinical experience	Median (range)
Years of practice	5.5 (1,41)
Intubations in the past year	2 (0,20)
Total career intubations	15 (0,100)
Ultrasound experience	
Currently using ultrasound	0/50
Previous ultrasound training	7/50
Previous airway ultrasound training	0/50

Baseline performance was 60% (30/50) for overall accuracy, 55% (12/22) for correct detection of an esophageal intubation, and 64% (18/28) for tracheal intubations. None of the scores showed significant differences when compared using a standard binomial test (Table 2). Post-test performance for correct esophageal intubation detection (96%, 25/26) and overall accuracy (82%, 41/50) were significant, with p-values <0.001. The post-test tracheal intubation score (66.7%, 16/24) did not show a significant difference compared to baseline performance.

**Table 2:** Accuracy of esophageal and tracheal intubation detection before and after training

	Pre-training	Post-training	McNemar’s p-value
Overall accuracy			
Correct, n (%)	30 (60)	41 (82)	
Incorrect, n (%)	20 (40)	9 (18)	
Correct (%) CI	45.2-73.6	68.6-91.4	
Total	50	50	
Binomial test p-value	0.20	<0.001	0.04 (50 paired)
Esophageal detection accuracy			
Correct, n (%)	12 (54.5)	25 (96.2)	
Incorrect, n (%)	10 (45.5)	1 (3.8)	
Correct (%) CI	32.2-75.6	80.4-99.9	
Total	22	26	
Binomial test p-value	0.83	<0.001	0.02 (11 paired)
Tracheal detection accuracy			
Correct, n (%)	18 (64.3)	16 (66.7)	
Incorrect, n (%)	10 (35.7)	8 (33.3)	
Correct (%) CI	44.1-81.4	44.7-84.4	
Total	28	24	
Binomial test p-value	0.19	0.15	0.62 (13 paired)

We found significant post-training increases in correct esophageal intubation identification performance as well as overall accuracy scores on all pre-test and post-test paired data using McNemar’s test, with p-values of 0.04 and 0.02, respectively. There was no meaningful change in paired correct tracheal detection scores.

The pre-test and post-test perceived usefulness scores were high and did not differ with respect to their median, 8 in both cases (Table 3), or through comparison according to the Wilcoxon signed-rank test.

**Table 3:** Before and after Likert ratings (1-10) of perceived usefulness of airway ultrasound

	Before training	After training
Median (range)	8 (1,10)	8 (3,10)
Completed	46	46
Missing	4	4
Wilcoxon p	0.07	

## Discussion

Participants demonstrated significant improvements in overall accuracy after a brief US training course. Post-test accuracy for esophageal intubation detection (96.2%) was particularly notable. As expected, baseline testing demonstrated no prior proficiency in airway US. Post-test scores in general were positive and slightly higher compared to those by Hanlin et al. who studied training in flight medics [7]. Results after training were similar to those of Lema et al., whose study protocol also included tube manipulation to facilitate correct position determination [8]. The post-test scores in overall accuracy (82%) and esophageal ETT detection (96%) after a short tutorial suggest that prehospital or hospital-based critical care providers who are US novices may benefit from this skill when the possibility of esophageal intubation is high and other detection devices are not readily available. Our prehospital participants experienced similar improvement after a rapid tutorial as emergency physicians and residents in an online education-based study by Chenkin et al. [2].

Correct detection of a tracheal intubation however remained unchanged following educational intervention. Additional training beyond a 15-minute course may be necessary for greater accuracy in identifying a tracheal intubation, as the structures in this view (ETT within the air-filled trachea) may be more difficult for participants to recognize compared to the double tract or "M" sign in the esophageal intubation views. Participants did comment that they felt more confident with the esophageal intubation views, as they felt the ETT was easier to identify on US when it was located in the esophagus versus the trachea. They reported that the esophageal intubation double tract sign appeared as a more readily visible distinct shape compared to the single air mucosa interface noted within the trachea as part of a tracheal intubation. The difference in view proficiency reflects trends noted in prior airway educational research that were not formally studied [7,8].

We note that our results could be due to the content and delivery of our specific training course. An additional limitation in our study design was the use cadavers rather than live patients. We acknowledge that US views in an intubated live patient may be different. Our sample size was noted to be small and limited to local participants. Analysis of long-term retention of knowledge and skill was not included in this study, and may be valuable in future research.

Although not formally tested, we additionally observed that many of our participants easily identified the triangular shape of the thyroid cartilage and curved shapes of the cricoid cartilage and tracheal rings on US during the instructional session. In critical situations where even a small amount of time is available to prepare for a necessary surgical airway (cricothyroidotomy), US may increase accuracy for providers, providing extra guidance for those who may hesitate when faced with this low-frequency but high-risk procedure. Further exploration of this use of US for those who perform emergency cricothyroidotomy may be worthwhile.

## Conclusions

The use of US to determine ETT placement is a skill that may be quickly acquired by novice learners who perform endotracheal intubation, likely due to the distinct views and superficial location of pertinent neck airway anatomy. Novice learners made notable proficiency gains specifically in the correct identification of an esophageal intubation after a brief training course, however they did not make significant progress in mastery of the tracheal intubation view. Increased emphasis on the more subtle findings that comprise the tracheal intubation US view may be useful in the educational design of airway US for novices.
